# Lexical-Semantic Variables Affecting Picture and Word Naming in Chinese: A Mixed Logit Model Study in Aphasia

**DOI:** 10.3233/BEN-2012-119002

**Published:** 2012-03-19

**Authors:** Davide Crepaldi, Wei-Chun Che, I.-Fan Su, Claudio Luzzatti

**Affiliations:** ^1^Department of PsychologyUniversity of Milano-BicoccaMilanItaly; ^2^Otorhinolaryngology DepartmentTaichung Veterans General HospitalTaichungTaiwan; ^3^Division of Speech and Hearing SciencesUniversity of Hong-KongHong-KongChina

**Keywords:** Chinese, aphasia, word naming, picture naming, imageability, age of acquisition, mixed-effect model, verbs, nouns

## Abstract

Lexical-semantic variables (such as word frequency, imageability and age of acquisition) have been studied extensively in neuropsychology to address the structure of the word production system. The evidence available on this issue is still rather controversial, mainly because of the very complex interrelations between lexical-semantic variables. Moreover, it is not clear whether the results obtained in Indo-European languages also hold in languages with a completely different structure and script, such as Chinese. The objective of the present study is to investigate this specific issue by studying the effect of word frequency, imageability, age of acquisition, visual complexity of the stimuli to be named, grammatical class and morphological structure in word and picture naming in Chinese. The effect of these variables on naming and reading accuracy of healthy and brain-damaged individuals is evaluated using mixed-effect models, a statistical technique that allows to model both fixed and random effects; this feature substantially enhances the statistical power of the technique, so that several variables–and their complex interrelations–can be handled effectively in a unique analysis. We found that grammatical class interacts consistently across tasks with morphological structure: all participants, both healthy and brain-damaged, found simple nouns significantly easier to read and name than complex nouns, whereas simple and complex verbs were of comparable difficulty. We also found that imageability was a strong predictor in picture naming, but not in word naming, whereas the contrary held true for age of acquisition. These results are taken to indicate the existence of a morphological level of processing in the Chinese word production system, and that reading aloud may occur along a non-semantic route (either lexical or sub-lexical) in this language.

## 1. Introduction

The study of lexical-semantic variables such as word frequency, imageability, and age of acquisition (AoA) has a long history in neuropsychological and cognitive research as a tool to inform models of lexical processing [26]. For example, the discovery that word frequency affects the time necessary for identifying a word [29], reading it aloud [3], or retrieving a word name after the presentation of a picture [51] triggered a vivacious and still vigorous debate on models of lexical selection [25,28,53,54]. Due to the strong intercorrelation of lexical-semantic variables [4,5], researchers have devoted substantial efforts in an attempt to disentangle the complex reciprocal relationships existing between word frequency, AoA, imageability, and morphological measures. For example, Lewis, Gerhand, and Ellis [41] provided evidence that both word frequency and AoA actually reflect a superordinate variable (cumulative frequency, i.e., the total number of times that a word has been encountered in life) and thus should not be considered as independent predictors of the behavior of brain-damaged and healthy individuals (see also [13]). The complex correlational structure of lexical-semantic variables has also been used to offer a direct cognitive interpretation of lexical effects: for example, based on the fact that frequency clustered

ISSN 0953-4180/12/$27.50 © 2012 – IOS Press and the authors. All rights reserved with semantic measures in their lexical decision experiment, Baayen, Feldman, and Schreuder [2] suggested that word frequency effects arise primarily at the semantic level, rather than being exclusively related to the frequency with which specific word forms are seen or heard. Another reason that cognitive scientists made thorough investigation into the role of lexical-semantic variables is that some of these correlate with other linguistic factors, such as grammatical class. Indeed, great efforts have been made by cognitive neuropsychologists to understand whether imageability could explain the difference in the performance of aphasic patients in typical naming tasks of nouns (highly imageable) and verbs (relatively less imageable) [11,12, 23,57]. In this context, Luzzatti, Raggi, Zonca, Pistarini, Contardi, and Pinna [46] demonstrated that the performance of some – but not all – aphasic patients apparently showing noun-verb dissociation in picture naming could be explained in terms of word frequency or imageability; these authors went on to show that imageability was the most relevant predictor in verb-impaired patients, whereas in noun-impaired patients word frequency played this role.

This vast literature is still far from clearly determining the role of each lexical-semantic variable; however, it has provided substantial neuropsychological and psycholinguistic evidence showing that word frequency, imageability, and AoA play a crucial role in determining the performance of brain-damaged and healthy individuals in a variety of tasks, including lexical decision, reading aloud, and picture naming.

However, the literature currently available has one shortcoming; lexical-semantic variables have been studied predominantly in alphabetical languages, and it is far from clear whether these results can be straightforwardly generalized to languages with completely different structures, like Chinese. Indeed, there are many reasons why Chinese and a Western language such as English may differ substantially from a cognitive point of view, particularly as far as the processing needed to convert orthographic symbols into an ordered sequence of phonemes (as in reading) is concerned.

Chinese script is often characterized as morphosyllabic because most of its basic entities (the characters) are monosyllabic and represent morphemes [24]. The basic features of written Chinese are the strokes, which are typically arranged in a squared pattern to form a character. Characters may vary substantially in their visual complexity (the number of strokes they contain ranges from 1 to 36) [44] and are typically composite, i.e., are made up of a semantic radical and a phonetic component. The semantic radical usually, but not always, provides an indication as to the semantic category of the character, whereas the phonetic component suggests its pronunciation; however, this is not always the case. Take for example the character 媽 (*/ma1/*- mother): it has the semantic radical 女 (*/nu3/*- woman) on the left and the phonetic component 馬 (*/ma3/*- horse) on the right; on the contrary, the character 猜 (*/cai1/* – to guess) is made up of the semantic radical 犭 (*/quan3/* – dog) – which is not related to the meaning of the whole character – and the phonetic component 青 (*/qing1/* – young, green, or blue), which bears no relationship to the sound of the character. Xing [76] estimates that around 25% of Chinese composite characters are pronounced exactly as their phonetic component, indicating that the phonetic component is not an effective cue to guess the pronunciation of the composite character. In Chinese, reliable print-to-sound correspondences cannot be established at the sub-component level either, as single strokes do not correspond to any phonemic unit. In addition, the tone of a character is not orthographically marked [38], thus highlighting again that proficient reading in Chinese must be heavily based on a lexical route.

In addition to its script, Chinese has other distinguishing features that are more general and thus likely to impact not only on orthographic identification and reading aloud, but also on other cognitive tasks such as lexical retrieval and naming. For example, unlike English and other Western languages, Chinese has very few inflectional and derivational morphemes and the morphological system is almost exclusively based on compounding, being the vast majority of the words morphologically complex; therefore the linguistic system of native speakers of Chinese might be closely bound to morphological analysis, which is not necessary in English or Dutch for example.

Despite these differences between Chinese and Western languages, the results that emerge from Chinese studies on lexical-semantic variables do not seem to differ substantially from those reported in studies on Indo-European languages. Bates et al. [8] and Zhang and Yang [73] found that word frequency is a predictor for the picture naming latency in healthy Chinese speakers (see also [44]). Weekes and colleagues [68], however, did not find significant impact of word frequency on picture naming latency, which is not uncommon in studies when other factors, such as AoA, are controlled [34,38]. When taking into consideration reading aloud, results are more clear-cut; as in other languages, characters with higher frequency are named more quickly and more accurately [33].

Several studies on Chinese, as those conducted in Western languages, have shown that words typically learned at a younger age are processed faster than words acquired later in life in a number of different tasks, including lexical decision on written words [15,16,64, 67], reading aloud [16,18,69], and semantic categorization [17,18,69]. It has also been suggested that AoA plays a role independently of word frequency both in lexical decision [64,67] and in reading aloud [34,38]. The effect of AoA has also been reported to influence aphasic patients' performance in reading aloud and picture naming [34,36,38]. In these studies, a patient (FWL) who suffered from severe semantic deficits was described; her condition was so severe that her word reading only relied on a non-semantic pathway. The authors also described a second patient (TWT) whose reading was clearly mediated by the semantic route as he made several semantic errors. They used a logistic regression analysis to investigate the ability of these two patients to read 260 characters aloud, and found that AoA was a significant predictor of the reading accuracy of both, indicating that AoA affects both the semantic and the non-semantic reading route. All the other variables that were considered in this study (e.g., character frequency, imageability, number of strokes, and semantic radical consistency) were not significant predictors. Although this study was seminal in considering several variables at the same time, it only focused on two brain-damaged individuals and did not consider healthy speakers, which hinders the generality of its results. Law et al. [36] investigated the picture naming performance of five anomic aphasic patients in a study where also object familiarity, naming agreement, visual complexity, and word length were considered; AoA turned out to be the strongest predictor.

Results of studies focusing on familiarity were much less clear-cut. Whereas some studies on Cantonese aphasic speakers report that familiarity does not play a role in picture naming accuracy [36], Weekes and colleagues [68] found that familiarity predicts picture naming reaction times in healthy speakers of Cantonese even after AoA is partialled out (see also [73]).

Studies exploring the number of strokes in a character as an indicator of visual complexity have also produced mixed results. Liu et al. [43] found that characters with fewer strokes were named faster, thus suggesting that the number of strokes in a character contributes significantly to naming speed in healthy speakers, but Law et al. [34,36] did not find a number of strokes effect in a word naming task performed by dyslexic readers.

Several studies have highlighted the importance of imageability in written lexical processing of Chinese and Kanji characters in Japanese. These studies focused on tasks as diverse as silent reading [32], recall of words [52], reading aloud [10], lexical decision [72], and semantic judgment on written words [61]. Some of these studies employed neurophysiological methodologies and showed imageability effects both in behavioral responses and in brain activity patterns. Notably, imageability correlates strongly with grammatical class, as nouns tend to be much more imageable than verbs, at least in picture naming; it is in fact no easy matter to disentangle these two effects. Zhang et al. [72] and Tsai et al. [61] provided solid evidence that imageability effects hold independently of grammatical class. In Zhang et al.'s study, for example, the imageability effect at the N400 was broader for nouns than for verbs as evidenced by the ERP topography. Tsai and colleagues [61] went on to show that concrete nouns and verbs elicit a greater N400 than abstract nouns and verbs in both lexical decision and semantic judgment tasks. Data are much less clear with regards to the impact of imageability on aphasic patients' behavior. For example, Bi et al. [10] reported an imageability effect in the reading performance of WJX, a patient who suffered from dementia. However, Law et al. [34] showed that, once other variables had been taken into consideration (e.g., AoA, character frequency, number of strokes), imageability was irrelevant for the reading performance of their two dyslexic patients (FWL and TWT).

To sum up, most of the lexical and lexical-semantic variables that have been shown to affect the performance of healthy and brain-damaged speakers in Western languages are also relevant in Chinese. Data are generally clearer on unimpaired individuals than on aphasic/dyslexic patients, most likely because the variables that best predict the performance of language impaired individuals may differ substantially depending on the specific cognitive impairment. What seems to be lacking is a study that takes this issue into consideration and thus focuses on a large group of brain-damaged individuals suffering from different types of aphasia, and differing widely on other dimensions, like lesion localization, deficit severity, age and education. In addition, most recent studies [2] have highlighted that the strong collinearity between lexical and lexical-semantic predictors makes it very difficult – perhaps impossible – to test a few of them without considering the others in the same design, which is another limitation of the studies conducted so far on Chinese; most of them, in fact, have focused on a small number of predictors (but see [34]). Finally, some variability has emerged in the various tasks that have been used in the literature, possibly reflecting the different cognitive levels they tap on. The core aim of the present study is therefore to address these problems:

(i) Addressing the role of several lexical-semantic variables simultaneously;
(ii) In two different tasks (picture and word naming) that require different cognitive processes;
(iii) In a large sample of healthy and aphasic speakers of different types.

As in the literature regarding Western languages, picture naming tasks were used to identify noun-verb dissociation in Chinese aphasic speakers [7,19]. Bates et al. [7] tested the noun-verb dissociation in Broca's and Wernicke's aphasic speakers of Chinese and reported that the former group performs better on object naming than action naming, whereas the contrary holds for the latter group. On the basis of these results, the authors suggested that nouns and verbs are represented differently at the lexical level in Chinese. Although lexical-semantic variables were not taken into consideration in the analyses of the data in this study, Chen and Bates [19] did provide additional evidence that grammatical class is likely to be an organizing principle of the lexical production system in Chinese. For this reason – and also to provide a further assessment of whether grammatical class explains speakers' performance over and above other lexical-semantic variables – the set of items for the present study will include both nouns and verbs and the data will be analysed also on the basis of grammatical class.

Although shown to be a strong determinant of behavior both in aphasic patients [8] and in healthy speakers [60], morphological structure has been somewhat neglected in the literature on Chinese. Interestingly, several psycholinguistic studies have been carried out in Chinese on written compound recognition [61,74,75], but much less attention has been paid to lexical production (see [50]). Chen and Chen [20] carried out implicit priming experiments where participants learned arbitrary associations between pairs of compound words, and were subsequently asked to produce one item of the pair after being cued with the other one. Response times were shown to be equivalent on pairs where compound words shared a morpheme in the initial position (e.g., 家事, *jia1-shi4*, household, and 家電, *jia1-dia4*, household appliances) and on pairs where compound words shared only a homophonic, non-homographic syllable in the same position (e.g., 家事, *jia1-shi4*, household, and 佳餚, *jia1-yao2*, delicacy). As morphological priming was equivalent to phonological priming in this experiment, the authors suggested that morphology is not an organizing principle of the word production system over and above phonology. This conclusion received further support from other experiments [20,31], showing that in a number of tasks (including picture naming) the frequency of the individual constituents does not influence the time necessary for producing a compound. This body of evidence is very intriguing because it seems to deny a level of morphological processing in a language where over 70% of words are compounds [75].

The role of morphology in Chinese is also debated in the literature on language and literacy acquisition. For example, McBride and colleagues [47,48] have shown that morphological awareness is associated with vocabulary knowledge in Chinese-speaking second graders, and also correlates with character recognition in preschoolers and second graders after controlling for age, phonological awareness, speed of processing, and vocabulary size. These results suggest that morphology contributes to language acquisition and the development of literacy skills over and above phonology. Sensitivity to the morphological structure of Chinese words was also found later in development among fourth-graders by Liu and colleagues [42]. However, Chung and Hu [21] have shown that morphological awareness is not associated to the ability to read Chinese characters once vocabulary knowledge had been partialled out; the authors concluded from these data that morphological knowledge in reading does not facilitate performance in the very initial stages of reading acquisition.

As we have illustrated, there seems to be substantial disagreement as to the role of morphology in the Chinese word identification and word production system. For this reason, we included both simple (i.e., monosyllabic, monomorphemic and one-character) and complex (bisyllabic, bimorphemic and two-character) words in our set of stimuli, and considered morphological structure as a further potential predictor of speakers' performance in our analyses.

## 2. Materials and methods

### *2.1. Participants*

Twenty Taiwanese speakers suffering from aphasia after a vascular left-hemisphere brain damage (12 suffering from Broca's aphasia, 2 from Wernicke's

Table 1
Examples of the types of items used in this paper

| Categories | Constituents | Example | Transcription and English translation |
|---|---|---|---|
| Simple noun | | 箭 | *jian4*, arrow |
| Simple verb | | 哭 | *ku1*, to cry |
| Nominal compound | | | |
| $[NN]_N$ | N+N | 奶瓶 | *nai3-ping2*, "milk + bottle", feeding-bottle |
| $[NV]_N$ | N+V | 沙漏 | *sa1-lou4*, "stand + to leak" hourglass |
| $[VN]_N$ | V+N | 剪刀 | *jian3-dao1*, "to cut + knife", scissors |
| $[VV]_N$ | V+V | 裁判 | *cai2-pan4*, "to judge + to decide" (the) referee |
| Verbal compound | | | |
| $[NV]_V$ | N+V | 肩負 | *jian1-fu4*, "shoulder + to carry something on the back", to bear |
| $[VN]_V$ | V+N | 拔河 | *ba2-he2,* "to pull out + river", to do a tug of war |
| $[VV]_V$ | V+V | 賽跑 | *sai4-pao3*, "to compete + to run", to race |

Notes. N, noun; V, verb.

aphasia, 3 from anomic aphasia, and 3 from a non-classifiable form of aphasia) were recruited for the study. Prior to brain damage they were proficient in Mandarin Chinese, which they used for everyday communication.[1] None suffered from severe dysarthria, severe apraxia of speech, auditory problems, visual problems, or more general cognitive impairments. All aphasic patients were at least six months post-onset. They participated in both a picture naming and a reading task, with the exception of participant A13, who could not complete the reading task. Twenty neurologically healthy individuals also participated in this study; they were matched in gender, age, and education level with the aphasic patients and were all proficient in Mandarin Chinese.

### 2.2. Materials

Two tasks – a picture naming and a reading task – were specifically designed to test the participants' ability to retrieve morphologically simple and complex nouns and verbs. Both tasks contained simple nouns, simple verbs, verbal compounds and nominal compounds. The items for nominal and verbal compounds were further divided into groups according to the grammatical category of their constituents. Nominal compounds were composed of a noun plus a noun ([NN]N), or a noun plus a verb ([NV]N), or a verb plus a noun ([VN]N), or a verb plus a verb ([VV]N). Verbal compounds were composed of a verb plus a verb ([VV]V), or a verb plus a noun ([VN]V), or a noun plus a verb ([NV]V). There were six categories and 95 items in total for the picture naming task: 20 simple nouns, 20 [NN]N, 20 [VN]N, 10 simple verbs, 15 [VN]V, and 10 [VV]V. Three categories, [NV]N, [VV]N, and [NV]V, were not included in the picture naming task because too few depictable testing items could be found. There were nine categories of stimuli in the reading task: simple nouns, [NN]N, [NV]N, [VN]N, [VV]N, simple verbs, [NV]V, [VN]V, and [VV]V) (see Table 1 for examples). Each category contained 20 items, for a total of 180 items for the whole task. [VN]V compounds are notoriously difficult to distinguish from verbal phrases. The criteria described by Packard [55] were adopted to define this type of verbal compounds in the present study. Verb+object elements (V-O) were thus considered as verbal compounds when:

(i) One of the constituents was a bound morpheme;
(ii) The V-O could be followed by an object;
(iii) The meaning of the V-O compound could not be inferred from the meaning of its constituents.

For the picture naming task, naming agreement was estimated for each item on the basis of the naming performance of 30 healthy participants, aged from 21 to 33. Only pictures whose naming agreement was above 70% were retained for the final version of the test: alternative answers that were given by at least 10% of the healthy participants were considered to be correct if produced by the aphasic patients. In order to avoid unnecessary collinearity among predictors, the word frequency, familiarity, imageability, and AoA of the items used in the picture naming and the reading aloud task were matched as closely as possible (see Table 2). Because no data are available on oral word frequency in Chinese, written frequency was considered in both the picture and the word naming task; this does not limit the generality of our findings because written and oral word frequency have been shown to correlate strictly [2]. Frequency values were obtained by con-

[1] Taiwanese is still very diffused in Taiwan, particularly among older people; therefore, some of the participants in this study also used Taiwanese extensively for everyday communication.

Table 2
Word frequency (*WF*), familiarity (*Fam*), imageability (*Img*), and age of acquisition (*AoA*) values for the different types of stimuli used in the present study (mean ± standard deviation)

| | | N | WF | Fam | Img | AoA |
|---|---|---|---|---|---|---|
| (a) Picture naming | | | | | | |
| Nouns | | | | | | |
| | Simple | 20 | 5.40 ± 0.93 | 5.37 ± 1.23 | 6.62 ± 0.32 | 2.32 ± 0.62 |
| | NN | 20 | 3.45 ± 0.94 | 5.10 ± 1.03 | 6.69 ± 0.17 | 3.02 ± 0.77 |
| | VN | 20 | 2.91 ± 1.50 | 4.92 ± 1.25 | 6.57 ± 0.30 | 3.23 ± 0.79 |
| Verbs | | | | | | |
| | Simple | 10 | 5.12 ± 1.45 | 5.63 ± 0.66 | 5.58 ± 0.56 | 2.38 ± 0.52 |
| | VN | 15 | 1.14 ± 1.14 | 4.92 ± 1.31 | 5.78 ± 0.49 | 3.40 ± 1.06 |
| | VV | 10 | 3.53 ± 0.69 | 4.73 ± 1.01 | 5.51 ± 0.60 | 3.96 ± 0.87 |
| (b) Reading aloud | | | | | | |
| Nouns | | | | | | |
| | Simple | 20 | 4.93 ± 0.69 | 5.03 ± 1.17 | 6.44 ± 0.63 | 2.56 ± 0.58 |
| | NN | 20 | 4.72 ± 1.07 | 4.63 ± 0.76 | 4.13 ± 2.06 | 4.51 ± 1.11 |
| | NV | 20 | 3.23 ± 1.61 | 4.42 ± 0.71 | 4.26 ± 1.88 | 5.15 ± 0.97 |
| | VN | 20 | 3.91 ± 1.69 | 4.92 ± 1.06 | 4.19 ± 1.96 | 4.43 ± 1.16 |
| | VV | 20 | 4.35 ± 1.45 | 4.69 ± 0.79 | 3.04 ± 1.40 | 5.15 ± 0.84 |
| Verbs | | | | | | |
| | Simple | 20 | 4.86 ± 1.08 | 5.36 ± 0.70 | 5.41 ± 0.88 | 2.41 ± 0.57 |
| | NV | 20 | 2.76 ± 0.93 | 3.64 ± 0.56 | 2.40 ± 1.03 | 5.85 ± 0.39 |
| | VN | 20 | 3.74 ± 1.58 | 4.74 ± 0.83 | 2.97 ± 0.90 | 4.99 ± 0.86 |
| | VV | 20 | 4.27 ± 1.36 | 4.58 ± 0.76 | 3.10 ± 1.08 | 4.86 ± 0.69 |

sulting the Academia Sinica Balanced Corpus of Modern Chinese (*http://www.sinica.edu.tw/SinicaCorpus*). The corpus is based on about 5 millions written words taken from various sources, such as newspapers, play scripts, and essays. Ratings of word familiarity and imageability were obtained by using a 7-point scale ranging from 1 (not familiar/imageable) to 7 (very familiar/imageable); for the imageability ratings, participants were asked to score each word according to the ease with which it evoked a mental image. The ratings of AoA were estimated on a 9-point scale: 1 corresponded to acquisition within the second year of life, 2 within the third year of life and so on until 9 (13 years of age or later). The ratings for each variable were made by at least 23 volunteer participants (age ranged from 19 to 33), none of which had participated in the naming agreement study. The number of strokes making up each character was also computed at this stage; this variable ranged from 4 to 20 in simple words (average = 11.95), and from 2 to 25 (for each character) in complex words (average = 10.62).

Certain words or characters occurred twice across the tests: in the picture naming task, one character was repeated twice among nouns, one was repeated twice among verbs, and 1 character was repeated twice across nouns and verbs. In the reading aloud task, 29 characters appeared twice. Overall, 18 characters were repeated across tasks, all among simple nouns and verbs. Specific care was thus taken to arrange the stimuli in separate sessions, so that none of the participants saw the same character twice in the same session (see below).

## 2.3. General procedures

Pictures and written words were shown one by one to the participants on a 15 × 20 cm paper sheet. Objects and actions were presented in two separate blocks in a semi-randomized order; the items with repeated characters were kept apart as much as possible. In the reading aloud task, nouns and verbs were instead tested together and were semi-randomized into two blocks, so that no repeated characters occurred in the same block. Participants were presented with a first block of the reading aloud task, then with the two blocks of picture naming task, and finally with a second block of the reading aloud task. The presentation order of the noun and verb blocks in the picture naming task was counterbalanced across subjects. The four testing sessions were carried out on different days for most patients. Healthy control speakers were tested following exactly the same procedure used with the aphasic patients, except that they were tested first on the picture naming blocks, and then on the word naming blocks. This was done in order to avoid repetition effects in the picture naming task on those items that were also included in the reading task; these effects were thought to have no impact on the reading task, as healthy speakers were expected to perform at ceiling in reading aloud, while the same assumption was not justified *a priori* for the picture naming task (as demonstrated by the imperfect naming agreement on several drawings).

In both tasks participants were given standard instructions ("please name the following pictures" or "please, read aloud the following words") followed by practice trials on words/pictures that were not included in the experimental sets. The tasks were administered in a quiet room by a speech and language pathologist (W-CC). Each session lasted about 45 minutes; participants could ask for a break at any time of the session. All the answers were recorded, transcribed, and scored after testing.

Responses were counted as correct only when participants responded appropriately and promptly, i.e., less than 3 seconds after the stimulus presentation. Taiwanese and Hakka dialects are still very common in Taiwan together with Mandarin Chinese, so target words named in either dialect were counted as correct.

### 2.4. Data analysis

Data were analyzed using Mixed Logit Models (MLM) [30]. MLM are similar to Logistic Regression Analysis (LRA) [49] because they study the relationship between several continuous or non-continuous independent predictors and one dichotomous dependent variable. However, MLM distinguish between *fixed effects*, i.e., effects that hold across the whole sample of patients, and *random effects*, i.e., patient-specific effects that are added to the fixed effects to provide a better account of the overall variability of the data. On the strength of this differentiation, MLM can address the question of whether any specific predictor has an impact on the performance of the whole sample of patients, as well as the question of whether patients differ in their sensitivity to this predictor.

MLM were fitted and analysed using the free statistical software R (version 2.10.1; *http://www.r-project.org/*), and in particular using the *lmer* function from the *lme4* package (*http://cran.r-project.org/web/packages/lme4/index.html*). The R code is available from the authors on request. Before fitting the models we analysed the correlational structure of the predictors and took the steps necessary to reduce collinearity (see below). An initial model was built up that included all main effects and second-level interactions as fixed effects; higher-level interactions were not considered because they seriously affect the sensitivity of the analyses of main effects and second-level interactions. This model also had a random intercept for subjects and for items; these effects are not related to any specific predictor, but account for the general variability related to the random selection of subjects (e.g., some people are generally more accurate than others) and items (e.g., some items are intrinsically more difficult than others). The initial model was then progressively simplified by removing stepwise non-significant fixed effects until the deletion of any additional effect caused a significant loss of fit to the model (as tested by a Chi-square test). Then the structure of the random effects specifically related to each predictor (*random slopes*) was examined, i.e., the parameters that indicate whether the effect of each specific predictor varies substantially across patients. The same stepwise procedure was applied here: each individual random effect was added to the model and its impact on the goodness of fit was tested. When the fit improved significantly, the specific random slope was retained in the model, otherwise it was removed. The analysis of the random slopes is also very useful because it captures variability that would be considered as error variance in standard regression or in ANOVA, thus limiting the sensitivity of the statistical test on fixed effects.

Grammatical class (nouns vs. verbs; *GC*), morphological structure (simple vs. complex; *Morph*), familiarity (*Fam*), age of acquisition (*AoA*), imageability (*Img*), and log-transformed word frequency (*WF*) were considered as possible predictors in the analysis of the healthy speakers' performance. Aphasia type (fluent vs. non-fluent vs. non-classified; *AT*) was added to the set of predictors for the analysis of the performance of the brain-damaged participants.

## 3. Results

### 3.1. Picture naming

#### 3.1.1. Correlation between predictors

The correlation matrix between the predictors in the picture naming task is shown in Table 3. A useful index to investigate the degree of collinearity among predictors is the condition number $k$ [9]. This index equals 16.46 in the matrix, thus indicating medium collinearity [1]. This can be attributed to the correlation between:

(i) *Img* and *GC* (nouns are more imageable than verbs);
(ii) *Morph* and *AoA* (simple words are judged to be learned earlier in life than complex words);

(iii) *Morph* and *WF* (simple words are more frequent than complex words);
(iv) *WF* and *AoA* (frequent words are judged to be learned earlier in life);
(v) *AoA* and *Fam* (words that are judged to be learned earlier in life are also judged as more familiar).

We tried to reduce collinearity by using factorial analysis, but no factorial solution was satisfying, i.e., factors were neither clearly interpretable theoretically nor allowed a consistent reduction of collinearity. We then tried to exclude the factors that were involved in the strongest correlations. The highest correlation index in the matrix is between *GC* and *Img*; however, we could not drop either of these variables because they clearly map onto separate theoretical concepts, both of which were of interest to us. We then turned our attention to the second strongest correlation in the matrix, which is between *AoA* and *Fam*. The theoretical constructs underlying these variables are not clearly distinguishable; no one can really remember as an adult when s/he has learned a specific word, and thus the subjective AoA ratings might reflect some sort of "introspective feeling of strength" about the representation of any given word, which might really be what Fam ratings are also based on. If this is the case, *Fam* and *AoA* are two different measures of the same construct: we thus felt that we could drop either of these variables without a significant loss of theoretical strength for our study. Fam was excluded rather than AoA because this latter variable has received substantial attention in the relevant literature and was thus more important to allow a meaningful comparison between our results and those obtained in past studies. The removal of *Fam* was sufficient for $k$ to drop to 6.62, indicating that the following analyses could be carried out safely [1].

### 3.1.2. Healthy participants

The overall average accuracy of the healthy participants is reported in Table 4 (upper part). Not all the participants performed at ceiling, particularly on verbs. The sub-optimal performance of the healthy speakers provided the opportunity of conducting a statistical analysis of the impact of the predictors on response accuracy. MLM analyses indicated that the speakers' performance was influenced by *GC*, *Morph*, *Img*, and by the joint effects of *GC* and *Morph* (see Table 5). In MLM, the *Beta* parameters indicate either a correlation between the predictor and the probability of success (if the predictor is continuous), or a change in probability of success with respect to a reference level (if the predictor is dichotomous). So, for example, the reference level for *GC* is noun; thus, the positive *Beta* for *GC* indicates that the probability of success is higher in verbs as compared to nouns.[2] Because the reference level for *Morph* is complex words, the positive *Beta* for this factor indicates that simple words are easier to name than complex words. The positive *Beta* for *Img* shows that high-imageability words are easier to name than low-imageability words. Since the reference levels for *GC* and *Morph* are nouns and complex words respectively, the interaction between these variables indicates a drop in probability of success (*Beta* is negative) when the word to be named is a verb and is morphologically simple; this suggests that the general advantage for simple over complex words revealed by the *Morph* main effect is less for verbs as compared to nouns (see Fig. 1 for a complete illustration of the *GC* × *Morph* interaction). Because no random slope determined a significant increase in the model goodness of fit, the fixed effects described above can be taken to be constant across subjects. The overall goodness of fit of the model, measured by the Somers' *Dxy*, is very satisfactory: this index quantifies the correlation between predicted and observed accuracy and equals 0.80 in the final model [1].

Table 3
Correlation matrix between the predictors in the picture naming task. Spearman's $r$ – rather than Pearson's $r$ – was used because morphological structure and grammatical class are dichotomous variables

| | GC | Morph | Fam | Img | AoA | WF |
|---|---|---|---|---|---|---|
| GC | 1 | | | | | |
| Morph | −0.05 | 1 | | | | |
| *Fam* | −0.04 | 0.20 | 1 | | | |
| *Img* | −0.78 | 0.03 | 0.31 | 1 | | |
| *AoA* | 0.20 | −0.49 | −0.73 | −0.41 | 1 | |
| *WF* | 0.02 | 0.64 | 0.45 | 0.00 | −0.53 | 1 |

Notes. *GC*, grammatical class; *Morph*, morphological structure; *Fam*, familiarity; *Img*, imageability; *AoA*, age of acquisition; *WF*, word frequency.

### 3.1.3. Brain-damaged patients

The overall average accuracy achieved by the brain-damaged participants in the picture naming task is reported in Table 4 (lower part) and shows that patients vary greatly in their pattern of performance. In certain patients (e.g., A01, A13), the picture naming ability is dramatically impaired, whereas others (e.g., A15, A18)

[2]This main effect of grammatical class is better qualified by the GC × Morph interaction that emerges in these analyses and in the subsequent MLMs.

Table 4
Mean accuracy (proportion of correct responses) shown by healthy speakers and brain-damaged patients in the picture naming task

| | | | | Nouns | | | Verbs | | | |
|---|---|---|---|---|---|---|---|---|---|---|
| Sbj ID | Age | Ed | Group | S | C | Tot | S | C | Tot | Grand Tot |
| C01 | 21 | 14 | Healthy | 1 | 0.95 | 0.97 | 1 | 1 | 1 | 0.98 |
| C02 | 36 | 12 | Healthy | 1 | 0.93 | 0.95 | 1 | 0.92 | 0.94 | 0.95 |
| C03 | 21 | 14 | Healthy | 0.95 | 0.95 | 0.95 | 0.90 | 0.96 | 0.94 | 0.95 |
| C04 | 30 | 16 | Healthy | 0.95 | 1 | 0.98 | 1 | 0.68 | 0.77 | 0.91 |
| C05 | 39 | 14 | Healthy | 1 | 0.98 | 0.98 | 1 | 0.84 | 0.89 | 0.95 |
| C06 | 42 | 14 | Healthy | 0.95 | 0.85 | 0.88 | 0.90 | 0.88 | 0.89 | 0.88 |
| C07 | 43 | 14 | Healthy | 1 | 0.88 | 0.92 | 0.80 | 1 | 0.94 | 0.93 |
| C08 | 42 | 16 | Healthy | 0.95 | 0.93 | 0.93 | 0.90 | 0.96 | 0.94 | 0.94 |
| C09 | 44 | 16 | Healthy | 1 | 0.95 | 0.97 | 1 | 0.96 | 0.97 | 0.97 |
| C10 | 51 | 16 | Healthy | 0.95 | 0.83 | 0.87 | 1 | 0.80 | 0.86 | 0.86 |
| C11 | 57 | 6 | Healthy | 1 | 0.70 | 0.80 | 0.80 | 0.60 | 0.66 | 0.75 |
| C12 | 57 | 16 | Healthy | 1 | 0.85 | 0.90 | 1 | 0.92 | 0.94 | 0.92 |
| C13 | 35 | 14 | Healthy | 1 | 0.95 | 0.97 | 1 | 0.84 | 0.89 | 0.94 |
| C14 | 84 | 12 | Healthy | 1 | 0.70 | 0.80 | 0.50 | 0.80 | 0.71 | 0.77 |
| C15 | 52 | 18 | Healthy | 0.95 | 0.80 | 0.85 | 0.80 | 0.68 | 0.71 | 0.80 |
| C16 | 59 | 8 | Healthy | 0.85 | 0.55 | 0.65 | 0.30 | 0.40 | 0.37 | 0.55 |
| C17 | 67 | 12 | Healthy | 0.90 | 0.73 | 0.78 | 0.80 | 0.80 | 0.80 | 0.79 |
| C18 | 36 | 16 | Healthy | 0.95 | 0.98 | 0.97 | 1 | 0.92 | 0.94 | 0.96 |
| C19 | 45 | 16 | Healthy | 1 | 0.80 | 0.87 | 1 | 0.76 | 0.83 | 0.85 |
| C20 | 42 | 14 | Healthy | 1 | 0.98 | 0.98 | 0.60 | 0.84 | 0.77 | 0.91 |
| A01 | 22 | 13 | AnF | 0.30 | 0.15 | 0.20 | 0.10 | 0.28 | 0.23 | 0.21 |
| A02 | 38 | 12 | AnF | 0.80 | 0.68 | 0.72 | 0.50 | 0.60 | 0.57 | 0.66 |
| A03 | 20 | 13 | AnF | 0.45 | 0.25 | 0.32 | 0.10 | 0.20 | 0.17 | 0.26 |
| A04 | 33 | 16 | AnF | 0.60 | 0.38 | 0.45 | 0.20 | 0.32 | 0.29 | 0.39 |
| A05 | 40 | 14 | AnF | 0.95 | 0.33 | 0.53 | 0.90 | 0.44 | 0.57 | 0.55 |
| A06 | 41 | 12 | AnF | 0.70 | 0.35 | 0.47 | 0.20 | 0.44 | 0.37 | 0.43 |
| A07 | 42 | 14 | AnF | 0.95 | 0.55 | 0.68 | 0.60 | 0.36 | 0.43 | 0.59 |
| A08 | 42 | 14 | AnF | 0.60 | 0.15 | 0.30 | 0.10 | 0.16 | 0.14 | 0.24 |
| A09 | 45 | 16 | AnF | 0.55 | 0.48 | 0.50 | 0.20 | 0.28 | 0.26 | 0.41 |
| A10 | 55 | 16 | AnF | 0.90 | 0.53 | 0.65 | 0.50 | 0.40 | 0.43 | 0.57 |
| A11 | 55 | 6 | AnF | 0.80 | 0.25 | 0.43 | 0.50 | 0.24 | 0.31 | 0.39 |
| A12 | 60 | 18 | AnF | 0.95 | 0.40 | 0.58 | 0.70 | 0.52 | 0.57 | 0.58 |
| A13 | 35 | 12 | AF | 0.40 | 0.15 | 0.23 | 0.10 | 0.08 | 0.09 | 0.18 |
| A14 | 79 | 16 | AF | 0.70 | 0.23 | 0.38 | 0.50 | 0.40 | 0.43 | 0.40 |
| A15 | 48 | 18 | AF | 0.95 | 0.75 | 0.82 | 0.50 | 0.76 | 0.69 | 0.77 |
| A16 | 65 | 6 | AF | 0.60 | 0.40 | 0.47 | 0.10 | 0.16 | 0.14 | 0.35 |
| A17 | 66 | 12 | AF | 0.85 | 0.33 | 0.50 | 0.60 | 0.44 | 0.49 | 0.49 |
| A18 | 40 | 16 | AnC | 0.90 | 0.83 | 0.85 | 0.70 | 0.88 | 0.83 | 0.84 |
| A19 | 42 | 16 | AnC | 0.95 | 0.70 | 0.78 | 0.70 | 0.64 | 0.66 | 0.74 |
| A20 | 45 | 14 | AnC | 0.85 | 0.28 | 0.47 | 0.70 | 0.32 | 0.43 | 0.45 |

*Notes. Ed*, education; *Healthy*, healthy speakers; *AF*, aphasic patient suffering from fluent aphasia; *AnF*, aphasic patient suffering from non-fluent aphasia; *AnC*, aphasic patient suffering from a form of aphasia that could not be classified; $S$, simple words; $C$, complex words.

show only mild impairment; some (e.g., A09, A16) perform very different on nouns and verbs, whereas others (e.g., A01, A12) behave similarly on the two word classes; some (e.g., A20) are very sensitive to the morphological structure of the target words, whereas others (e.g., A09) are not. However, as this paper focuses specifically on the role of lexical-semantic variables, our attention was concentrated on the MLM analyses.

The final model described in Table 6 shows that the patients' performance mainly depends on grammatical class, morphological structure, imageability, spoken word frequency, aphasia type, and on the joint effect of grammatical class and morphological structure. Regarding main effects, it was seen that:

(i) Verbs have a higher probability of being retrieved correctly than nouns;
(ii) Simple words have a higher probability of success than complex words;
(iii) High-imageability words are easier than low-imageability words;
(iv) *WF* correlates positively with probability of success;

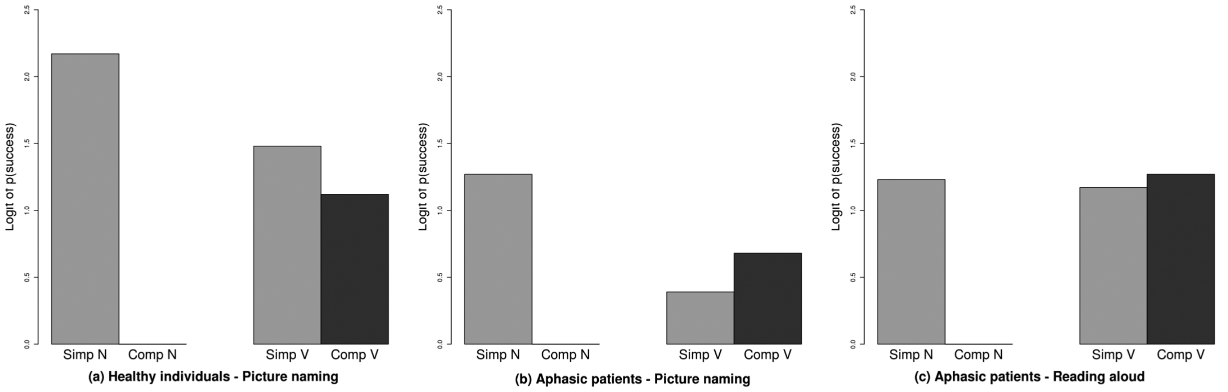

Fig. 1. Schematic representation of the *GC* × *Morph* interaction that emerged in the analysis of the performance of (a) healthy speakers in picture naming, (b) aphasic patients in picture naming, (c) aphasic patients in reading aloud. The performance of normal speakers in the reading task is not included in the figure because it was virtually at ceiling. Change in probability of success as measured against a reference level – i.e., complex nouns – is represented on the Y axis. This variable is calculated on the basis of the *Beta* parameters estimated by the MLM; this ensures that figures refer to the genuine effects of *GC* and *Morph*, i.e., once the contribution of all other predictors has been taken out. Because *Beta* parameters are additive, figures for each class are calculated by simply adding the relevant Betas; for example, the value for simple nouns is obtained by adding the parameter for simple words (*Beta*(*Morph*) = 2.17) to the parameter for nouns (0, because nouns are the reference level for the variable *GC*); similarly, the value for complex verbs is calculated by adding the parameter for complex words (0, because complex words are the reference level for the variable *Morph*) and the parameter for verbs (*Beta*(*GC*) = 1.12). Probability of success is expressed in the logit space; higher values mean higher probability of success.

Table 5

MLM offering the best fit to the observed performance of healthy speakers in the picture naming task

| Fixed effects | Beta | Std. error | z value | p |
|---|---|---|---|---|
| *Intercept* | −8.06 | 2.61 | −3.08 | 0.002 |
| *GC* | 1.12 | 0.54 | 2.07 | 0.04 |
| *Morph* | 2.17 | 0.55 | 3.95 | < 0.001 |
| *Img* | 1.62 | 0.39 | 4.14 | < 0.001 |
| *GC* × *Morph* | −1.81 | 0.78 | −2.33 | 0.02 |
| Random effects | Variance | | | |
| *Sbj* (*intercept*) | 1.04 | | | |
| *Item* (*intercept*) | 1.52 | | | |
| *Log Likelihood* = *−563.4* | | *Dxy* = *0.80* | | |

Notes. *GC*, grammatical class; *Morph*, morphological structure; *Img*, imageability. Reference levels are nouns for *GC* and complex words for *Morph*. *Dxy* refers to Somer's rank correlation between predicted probabilities and observed responses; this index varies from 0 (the model has no predictive value) to 1 (the model predicts the data perfectly).

Table 6

MLM offering the best fit to the observed performance of brain-damaged speakers in the picture naming task

| Fixed effects | Beta | Std. error | z value | p |
|---|---|---|---|---|
| *Intercept* | −7.58 | 1.71 | −4.47 | < 0.001 |
| *GC* | 0.68 | 0.33 | 2.04 | 0.04 |
| *Morph* | 1.27 | 0.37 | 3.45 | < 0.001 |
| *Img* | 0.90 | 0.25 | 3.66 | < 0.001 |
| *WF* | 0.29 | 0.08 | 3.57 | < 0.001 |
| *AT* (*non-fluent*) | 0.04 | 0.49 | 0.08 | 0.94 |
| *AT* (*non-classified*) | 1.37 | 0.69 | 2.06 | 0.04 |
| GC × Morph | −1.56 | 0.44 | −3.56 | < 0.001 |
| Random effects | Variance | Correlation | | |
| *Item* (*intercept*) | 0.57 | | | |
| *Sbj* (*intercept*) | 0.81 | | | |
| *Sbj* (*Morph slope*) | 0.71 | −0.20 | | |
| *Log Likelihood* = −1050 | | | *Dxy* = 0.70 | |

Notes. *GC*, grammatical class; *Morph*, morphological structure; *Img*, imageability; *WF*, log-transformed word frequency. Reference levels are nouns for *GC*, complex words for *Morph*, and fluent aphasic patients for *AT*. *Dxy* refers to Somer's rank correlation between predicted probabilities and observed responses; this index varies from 0 (the model has no predictive value) to 1 (the model predicts the data perfectly).

(v) Non-fluent patients were as compromised as fluent patients (*Beta* for *AT* (*non-fluent*) is non-significant), whereas non-classified patients had a better overall performance than fluent patients (*Beta* for *AT* (*non-classified*) is significant and positive).

In the brain-damaged participants, the interaction between *GC* and *Morph* indicates that the probability of success decreases for simple verbs (*Beta* is negative and the reference levels are nouns and complex words as above); this shows that the difference between simple verbs and complex verbs is less than the difference between simple nouns and complex nouns. It is interesting to note that the last two fixed effects removed from the model were AT × *GC* and *AT* × *Img*. Although they do not contribute significantly to the model fit, these effects were close to significance before being removed (*Beta* = −0.63; $z = -1.48$; $p = 0.14$ for *AT* × *GC*; *Beta* = −0.66; $z = -1.84$; $p = 0.06$ for *AT* × *Img*), indicating that non-fluent patients were less suc-

Table 7
Correlation matrix between the predictors in the reading task. Spearman's $r$ – rather than Pearson's $r$ – was used because morphological structure and grammatical class are dichotomous variables

| | GC | Morph | Fam | Img | AoA | WF |
|---|---|---|---|---|---|---|
| *GC* | 1 | | | | | |
| *Morph* | 0.06 | 1 | | | | |
| *Fam* | −0.06 | 0.30 | 1 | | | |
| *Img* | −0.24 | 0.54 | 0.28 | 1 | | |
| *AoA* | 0.06 | −0.69 | −0.55 | −0.76 | 1 | |
| *WF* | −0.12 | 0.30 | 0.46 | −0.02 | −0.30 | 1 |

Notes. *GC*, grammatical class; *Morph*, morphological structure; *Fam*, familiarity; *Img*, imageability; *AoA*, age of acquisition; *WF*, word frequency.

cessful in naming verbs than nouns (*Beta* was negative on $AT \times GC$) as well as in naming high-imageability words than low-imageability words (*Beta* was negative on $AT \times Img$). Quite surprisingly, no random slope was necessary for *GC* ($Chi^2$ between the model including this effect and the model without this effect is 0.89 on 2 degrees of freedom; $p = 0.64$), *Img* ($Chi^2 = 0.69$; $df = 3$; $p = 0.88$), and *WF* ($Chi^2 = 0.52$; $df = 3$; $p = 0.92$). The sensitivity shown to these factors by individual patients did not vary substantially within the participant sample. On the contrary, the introduction of a random slope for *Morph* in the model determined an increase in the model goodness of fit ($Chi^2 = 19.95$; $df = 2$; $p < 0.001$), showing that some patients – but not all – were sensitive to the morphological structure of words (some patients were better at naming simple words than complex words; e.g., A05, A12, and A20).

The overall goodness of fit of the model was quite good for the brain-damaged speakers too, as indicated by the fact that predicted and observed values correlate 0.70 (see the *Dxy* index in Table 4).

## 3.2. Reading aloud

### 3.2.1. Correlation between predictors

The correlation matrix between the predictors in the reading task in shown in Table 7 and is quite similar to that observed in the picture naming task. The most relevant differences are that *GC* and *Img* entertained a much weaker correlation (as stimuli did not need to be depicted, and so low-imageability nouns could be introduced into the battery), whereas *AoA* and *Img* are more strongly correlated (most imageable words are acquired earlier) in the reading task than in the picture naming task. As the theoretical constructs underlying *AoA* and *Img* are quite different, and both variables have been reported as important predictors in reading performance [2,41], neither were excluded from the subsequent analyses. *Fam* was excluded, as it was for the picture naming task, because it correlates strongly with both *AoA* and *WF*. The condition number $k$ [9] was 25.24 in the final set of predictors, thus indicating the existence of some collinearity, which, however, is not high enough to hinder the reliability of the MLM [1].

### 3.2.2. Healthy participants

The performance of the healthy participants in the reading task is described in Table 8 (upper part). Unlike the picture naming task, nearly all healthy participants performed at ceiling level in the reading aloud task. It is important to note that this was not due to a sampling bias; target words had comparable lexical-semantic characteristics in the two tasks (see above) given all other constraints (e.g., naming agreement). This asymmetry is most likely due to a particular feature of Chinese, in that pictures may be generally named through more alternative lexical labels [8] than in Western languages, and are thus more likely to elicit non-standard responses, particularly from the elderly and/or less educated. Critically, the fact that the performance of the healthy speakers was at ceiling in reading, but not in picture naming, does not affect the reliability and generality of our findings; subject-specific variability is absorbed by random effects in MLM, and thus the evaluation of the more general fixed effects is not compromised by this additional variance. One unfortunate aspect of the healthy speakers being at ceiling was that it was not possible to run MLM on their performance and so it was impossible to compare the impact of lexical-semantic variables on reading in healthy vs. brain-damaged participants.

### 3.2.3. Brain-damaged patients

The overall average accuracy of the brain-damaged participants in the reading task is reported in Table 8 (lower part). The final model is described in Table 9, and shows that:

(i) Verbs were marginally easier than nouns (*Beta* is positive, but just outside the significance threshold);
(ii) Simple words were read better than complex words;
(iii) *AoA* correlated positively with probability of success, but the effect is only marginally significant;
(iv) High-frequency words were more likely to be read correctly than low-frequency words;
(v) AT had no role in the prediction of accuracy;

Table 8
Mean accuracy shown by healthy speakers and brain-damaged patients in the reading aloud task

| | | | | Nouns | | | Verbs | | | |
|---|---|---|---|---|---|---|---|---|---|---|
| Sbj ID | Age | Ed | Group | S | C | Tot | S | C | Tot | Gran Tot |
| C01 | 21 | 14 | Healthy | 1 | 0.99 | 0.99 | 1 | 1 | 1 | 0.99 |
| C02 | 36 | 12 | Healthy | 1 | 0.99 | 0.99 | 1 | 1 | 1 | 0.99 |
| C03 | 21 | 14 | Healthy | 1 | 1 | 1 | 1 | 0.97 | 0.98 | 0.99 |
| C04 | 30 | 16 | Healthy | 1 | 1 | 1 | 1 | 1 | 1 | 1 |
| C05 | 39 | 14 | Healthy | 1 | 1 | 1 | 1 | 1 | 1 | 1 |
| C06 | 42 | 14 | Healthy | 1 | 1 | 1 | 1 | 1 | 1 | 1 |
| C07 | 43 | 14 | Healthy | 1 | 0.99 | 0.99 | 1 | 0.97 | 0.98 | 0.98 |
| C08 | 42 | 16 | Healthy | 1 | 1 | 1 | 1 | 1 | 1 | 1 |
| C09 | 44 | 16 | Healthy | 1 | 1 | 1 | 1 | 1 | 1 | 1 |
| C10 | 51 | 16 | Healthy | 1 | 1 | 1 | 1 | 0.98 | 0.99 | 0.99 |
| C11 | 57 | 6 | Healthy | 1 | 0.97 | 0.97 | 1 | 0.98 | 0.99 | 0.98 |
| C12 | 57 | 16 | Healthy | 1 | 0.99 | 0.99 | 1 | 1 | 1 | 0.99 |
| C13 | 35 | 14 | Healthy | 1 | 1 | 1 | 1 | 1 | 1 | 1 |
| C14 | 84 | 12 | Healthy | 1 | 0.99 | 0.99 | 1 | 0.98 | 0.99 | 0.99 |
| C15 | 52 | 18 | Healthy | 1 | 1 | 1 | 1 | 1 | 1 | 1 |
| C16 | 59 | 8 | Healthy | 1 | 0.98 | 0.98 | 1 | 0.97 | 0.98 | 0.98 |
| C17 | 67 | 12 | Healthy | 0.95 | 0.97 | 0.96 | 1 | 0.93 | 0.95 | 0.96 |
| C18 | 36 | 16 | Healthy | 1 | 0.98 | 0.98 | 1 | 0.98 | 0.99 | 0.98 |
| C19 | 45 | 16 | Healthy | 1 | 0.98 | 0.98 | 1 | 0.95 | 0.96 | 0.97 |
| C20 | 42 | 14 | Healthy | 1 | 1 | 1 | 1 | 0.95 | 0.96 | 0.98 |
| A01 | 22 | 13 | AnF | 0.95 | 0.54 | 0.62 | 0.95 | 0.53 | 0.63 | 0.63 |
| A02 | 38 | 12 | AnF | 0.90 | 0.69 | 0.73 | 0.85 | 0.65 | 0.70 | 0.72 |
| A03 | 20 | 13 | AnF | 0.85 | 0.31 | 0.42 | 0.80 | 0.27 | 0.40 | 0.41 |
| A04 | 33 | 16 | AnF | 1 | 0.78 | 0.82 | 1 | 0.82 | 0.86 | 0.84 |
| A05 | 40 | 14 | AnF | 0.95 | 0.88 | 0.89 | 0.90 | 0.87 | 0.88 | 0.88 |
| A06 | 41 | 12 | AnF | 0.90 | 0.87 | 0.87 | 0.90 | 0.83 | 0.85 | 0.86 |
| A07 | 42 | 14 | AnF | 1 | 0.73 | 0.78 | 0.95 | 0.67 | 0.74 | 0.76 |
| A08 | 42 | 14 | AnF | 0.80 | 0.12 | 0.25 | 0.55 | 0.12 | 0.23 | 0.24 |
| A09 | 45 | 16 | AnF | 0.75 | 0.65 | 0.67 | 0.60 | 0.77 | 0.73 | 0.69 |
| A10 | 55 | 16 | AnF | 0.95 | 0.95 | 0.95 | 1 | 0.95 | 0.97 | 0.96 |
| A11 | 55 | 6 | AnF | 0.95 | 0.81 | 0.84 | 0.95 | 0.82 | 0.85 | 0.84 |
| A12 | 60 | 18 | AnF | 0.95 | 0.66 | 0.72 | 0.90 | 0.53 | 0.63 | 0.68 |
| A13 | 35 | 12 | AF | NA | NA | NA | NA | NA | NA | NA |
| A14 | 79 | 16 | AF | 1 | 0.81 | 0.85 | 0.95 | 0.80 | 0.84 | 0.85 |
| A15 | 48 | 18 | AF | 1 | 1 | 1 | 1 | 0.97 | 0.98 | 0.99 |
| A16 | 65 | 6 | AF | 1 | 0.86 | 0.89 | 0.95 | 0.85 | 0.88 | 0.88 |
| A17 | 66 | 12 | AF | 1 | 0.86 | 0.89 | 0.85 | 0.87 | 0.86 | 0.88 |
| A18 | 40 | 16 | AnC | 0.95 | 0.98 | 0.97 | 1 | 1 | 1 | 0.98 |
| A19 | 42 | 16 | AnC | 0.95 | 0.91 | 0.92 | 0.90 | 0.92 | 0.91 | 0.92 |
| A20 | 45 | 14 | AnC | 0.90 | 0.44 | 0.53 | 0.80 | 0.38 | 0.49 | 0.51 |

Notes. *Ed*, education; *Healthy*, healthy speakers; *AF*, aphasic patient suffering from fluent aphasia; *AnF*, aphasic patient suffering from non-fluent aphasia; *AnC*, aphasic patient suffering from a form of aphasia that could not be classified; *S*, simple words; *C*, complex words; NA, not available.

(vi) The advantage of simple over complex words was higher in nouns than in verbs (as in the picture naming task, *Beta* for *GC* × *Morph* is positive and once again the reference levels are nouns and complex words);

(vii) The effect of AoA is weaker in verbs than in nouns (*Beta* for *GC* × *AoA* is negative), although this effect is only marginally significant;

(viii) *AoA* interacts with *WF*, indicating that words with high *AoA* and *WF* have lower probability of success;

(ix) *WF* has reduced impact on the performance of non-fluent and non-classified patients compared to fluent patients.

The goodness of fit of the model benefits from the addition of a random slope for *Morph* ($Chi^2 = 25.34$, $df = 2$, $p < 0.001$), thus indicating that patients differ in their sensitivity to morphological structure. Also, the random slopes for *AoA* and *WF* improve the model fit, but not significantly so (*AoA*: $Chi^2 = 4.70$, $df = 3$, $p = 0.20$; *WF*: $Chi^2 = 3.87$, $df = 3$, $p = 0.28$). On the contrary, there is no evidence at all for the insertion of random slopes for either *GC* or *GC* × *Morph*; patients

Table 9
MLM offering the best fit to the observed performance of brain-damaged speakers in the reading task

| Fixed effects | Beta | Std. error | z value | p |
|---|---|---|---|---|
| *Intercept* | 0.03 | 0.99 | 0.03 | 0.97 |
| *GC* | 1.27 | 0.76 | 1.68 | 0.09 |
| *Morph* | 1.23 | 0.39 | 3.12 | 0.001 |
| *AoA* | 0.28 | 0.15 | 1.81 | 0.07 |
| *WF* | 0.76 | 0.19 | 4.09 | $<0.001$ |
| *AT* (*non-fluent*) | −0.39 | 0.76 | −0.51 | 0.61 |
| *AT* (*non-classified*) | 0.12 | 1.00 | 0.12 | 0.90 |
| *GC* × *Morph* | −1.33 | 0.51 | −2.63 | 0.008 |
| *GC* × *AoA* | −0.26 | 0.15 | −1.77 | 0.07 |
| *AoA* × *WF* | −0.10 | 0.04 | −2.92 | 0.003 |
| *WF* × *AT* (*non-fluent*) | −0.25 | 0.09 | −2.79 | 0.005 |
| *WF* × *AT* (*non-classified*) | −0.23 | 0.12 | −1.96 | 0.04 |
| Random effects | Variance | | | |
| *Item* (*intercept*) | 0.18 | | | |
| *Sbj* (*intercept*) | 1.87 | | | |
| *Sbj* (*Morph slope*) | 0.79 | | | |
| *Log Likelihood* $= -1418$ | | | *Dxy* $= 0.70$ | |

Notes. *GC*, grammatical class; *Morph*, morphological structure; *AoA*, age of acquisition; *WF*, word frequency. Reference levels are nouns for *GC*, complex words for *Morph*, and fluent patients for *AT*. *Dxy* refers to Somer's rank correlation between predicted probabilities and observed responses; this index varies from 0 (the model has no predictive value) to 1 (the model predicts the data perfectly).

are thus quite homogeneous regarding these factors. The final model has a satisfactory predictive power as shown by the fact that Somer's *Dxy* $= 0.70$.

### 3.3. *Separate analyses on simple and complex nouns and verbs*

The MLM analyses described above show consistent effects of grammatical class, morphological structure, and an interaction between these variables. In order to investigate this interaction more in depth, separate MLM analyses were carried out on (a) simple nouns, (b) complex nouns, (c) simple verbs, and (d) complex verbs, in both picture naming and reading. Because the effects of *GC*, *Morph*, and *GC* × *Morph* were found in the healthy participants as well as in brain-damaged patients in the previous analyses on picture naming, data from these two populations were analyzed jointly.

In the subsequent analyses on the picture naming task, the starting model included *Group* (brain-damaged individuals – which is the reference level – vs. healthy speakers), *AoA*, *Img*, *WF*, and the interaction between *Group* and these three latter variables as fixed effects. The grammatical class of the constituents (*ConstGC*; noun-noun vs. noun-verb vs. verb-noun vs. verb-verb) was also included in the analyses of the performance on compound words. Random intercepts for items and subjects were included in the initial model. The starting model was identical with that used for the reading aloud data, except that the analyses were carried out on the aphasic speakers only, and thus *Group* was not among the predictors. Moreover, an index of the visual complexity of the characters to be read (i.e., the number of strokes they are composed of) was also included in the reading aloud analyses.

#### 3.3.1. *Picture naming*

*Simple nouns*

The final MLM included *AoA* (*Beta* $= -0.91$; $z = -2.37$; $p = 0.02$), *WF* (*Beta* $= 0.30$; $z = 1.52$; $p = 0.13$), *Group* (*Beta* $= 7.73$; $z = 3.20$; $p = 0.001$), and the interaction between this latter factor and *AoA* (*Beta* $= -1.70$; $z = -2.33$; $p = 0.02$) as fixed effects; moreover, the model included a random slope for *AoA*, showing that participants differ in their sensitivity to this factor. This model indicates that the probability of success for simple nouns:

(i) Increases as *AoA* decreases, even if this effect is less evident in neurologically intact speakers;
(ii) Is only marginally higher for high-frequency compared to for low-frequency words;
(iii) Is higher in healthy individuals than in brain-damaged participants.

*Simple verbs*

The final MLM for simple verbs in picture naming included only two fixed effects: *Group* (brain-damaged individuals vs. healthy participants; *Beta* $= 3.13$; $z = 5.740$; $p < 0.001$) and *AoA* (*Beta* $= -1.27$; $z = -3.23$; $p = 0.001$). Not surprisingly, this indicates that healthy participants performed better than brain-damaged individuals, and that words learnt early in life were the easiest to retrieve overall. No random slope determined a significant increase in the goodness of fit of the model.

*Complex nouns*

Due to the constraints posed on the item selection, complex nouns only included noun-noun and verb-noun compounds; the variable *ConstGC* thus included these two levels only (with noun-noun compounds taken as the reference level). The final model included *Group* (*Beta* $= -5.84$; $z = -1.52$; $p = 0.12$), *Img* (*Beta* $= 1.76$; $z = -3.23$; $p = 0.001$), *WF* (*Beta* $= 0.28$; $z = 1.99$; $p = 0.04$), and *Group* × *Img* (*Beta* $= 1.37$; $z = 2.36$; $p = 0.02$) as fixed effects, and no additional random slopes. Interestingly, the grammatical class of the constituents did not play any role in complex noun retrieval.

*Complex verbs*

Items in this category included verb-noun and verb-verb compounds; the former group constituted the reference level for the variable *ConstGC*. The final model included Group (*Beta* $= 2.67$; $z = 8.12$; $p < 0.001$), *ConstGC* (*Beta* $= -0.42$; $z = -1.43$; $p = 0.15$), *Img* (*Beta* $= 0.51$; $z = 1.53$; $p = 0.12$), and *AoA* (*Beta* $= -0.36$; $z = -2.19$; $p = 0.03$) as fixed effects, and a random slope for *Img*. This model shows that healthy participants performed better than brain-damaged individuals. It also indicates that performance was slightly better on verb-noun compounds as opposed to on verb-verb compounds, and confirms the effect of imageability observed on complex nouns, even if this effect did not interact with participant group in this analysis.

### 3.3.2. Reading aloud

Only data regarding the reading aloud performance of the brain-damaged participants were analysed as all healthy participants performed at ceiling.

*Simple nouns*

The final MLM included only the intercept as a fixed effect; there was no statistic justification for introducing any of the predictors into the model as none determined a significant improvement of the goodness of fit. The final model did not include any random slope. This produced a rather unusual MLM, which might be partially attributed to the fact that the brain-damaged patients too performed close to ceiling on simple nouns (the proportion of correct responses varied from 0.75 to 1; median = 0.95; see Table 6).

*Simple verbs*

The final MLM included *Img* (*Beta* $= -0.59$; $z = -2.06$; $p = 0.04$) and *AoA* (*Beta* $= -1.11$; $z = -3.06$; $p = 0.002$) as fixed effects; the absence of a random slope produced a significant increase in the model goodness of fit. Interestingly, the negative *Beta* for this *Img* indicates that the performance of brain-damaged individuals on simple verbs increases as imageability decreases (reverse imageability effect). However, caution must be used when interpreting the results of this MLM analysis because the reading performance was nearly at ceiling on simple verbs (range of proportion correct = 0.55–1; median = 0.925; see Table 6).

*Complex nouns*

The final MLM only included the fixed-effect of the number of strokes of the first constituent (*Beta* $= -0.04$; $z = -2.03$; $p = 0.04$) and the random intercepts for items and subjects; no lexical-semantic predictor determined a significant increase in the goodness of fit of the model. Thus, the performance of the brain-damaged participants on complex noun reading was unaffected by the grammatical class of the constituents, imageability, AoA, and frequency. The overall goodness of fit of the model improved when a random slope for *Img* was included into the model, thus showing cross-subject variability for sensitivity to this factor.

*Complex verbs*

The final MLM fit to these data included *AoA* as a fixed effect (*Beta* $= -0.37$; $z = -2.81$; $p = 0.004$), but no random slopes. It is worth noting that written frequency was close to being significant (*Beta* $= 0.10$; $z = 1.56$; $p = 0.12$) before being excluded from the model; moreover, its contribution to the model goodness of fit was not entirely negligible – although non-significant ($Chi^2$ between the model including this effect and the model without this effect is 2.34 on 1 degree of freedom; $p = 0.13$). As for the simple words – and contrary to the nominal compounds –, the number of strokes making up the characters does not seem to influence the patients' performance in word naming.

## 4. Discussion

The objective of the present study is to investigate the impact of lexical-semantic variables on picture and word naming in healthy and aphasic Chinese speakers, with a particular focus on the role of written word frequency, familiarity, age of acquisition (AoA), imageability, morphological structure, and grammatical class. Five main findings emerged:

(i) An interaction exists between grammatical class and morphological structure in both tasks and in both groups of participants, indicating that complex nouns were far more difficult to retrieve than simple nouns, but the effect of complexity was greatly reduced (or absent) in verbs;
(ii) The effect of morphological complexity varied substantially across the sample of patients in both tasks, as indicated by the by-subject random slope for morphological structure in the relevant Mixed Logit Models (*MLM*);
(iii) Imageability was a significant predictor of picture naming accuracy in both healthy and aphasic speakers, whereas it did not predict either the patients' or the healthy participants' performance in word naming;
(iv) Word frequency was a significant predictor in both picture and word naming, but only for the aphasic participants;
(v) Finally, AoA contributes to the explanation of the patients' performance in the word naming task, but not in the picture naming task.

### 4.1. Morphology and grammatical class

As illustrated in the Introduction, some results suggest minimal involvement of morphological encoding in the lexical production of Chinese [20,31], which is very interesting considering the extreme productivity of compounding in this language. The results obtained in the present study are clearly in conflict with Chen et al.'s [20] and Janssen et al.'s [31] results. Retrieval of simple words, at least for nouns, was consistently better than that of complex words. This might be attributed to an effect of difficulty, but certain considerations suggest otherwise:

(i) In the present study, the effect of morphological structure emerged independently of word frequency, imageability, AoA, and other lexical-semantic variables (which were taken into account independently in the MLM);
(ii) The interaction between morphology and grammatical class was very consistent (i.e., in both tasks and in both healthy and brain-damaged participants); this is difficult to explain if one considers morphological effects just as due to difficulty.

Intriguingly, evidence for morphological decomposition is available in the literature on Chinese word recognition. However, the morphological effects described in this paper cannot be interpreted as being due to the word recognition system because they also emerge in picture naming, in which no written word identification process is involved. Therefore, data seem to point to a morphological level of representation in the Chinese word production system, in analogy to what has been suggested for Indo-European languages [39, 45].

How can the present data be reconciled with the lack of morphological effects in Chen and Chen's [20] and Janssen et al.'s [31] studies? One possibility is that these experiments may have failed to detect morphological effects in spite of the existence of a morphological level of representation in the Chinese word production system. In Chen and Chen's [20] experiment, for example, participants were trained to associate cue and target words that were semantically related in the vast majority of cases; the morphological effect was thus likely to add on a baseline semantic effect, which may have made morphological priming more difficult to detect. In line with this hypothesis, the morphological facilitation highlighted by Chen and Chen [20] was indeed greater than the phonological facilitation, but this difference fell short of reaching significance (Experiment 3: $p = 0.16$ in the by-subject analysis). As far as the lack of morpheme frequency effects in picture naming [31] and in Chen and Chen's [20] task is concerned, results indicate the absence of a morphological level of representation *only if* the morpheme frequency effect and the whole-word frequency effect are assumed to be *additive*. In an interactive system where a morphological level of representation exists, but morpheme and whole-word selection overlap in time and influence each other, it might well be the case that word frequency effects hide morpheme frequency effects, or vice versa. Taft [59] demonstrated this point elegantly in a lexical decision experiment. Using the same experimental items, he showed both equivalent and completely opposite effects of morpheme and whole-word frequency by manipulating the filler trials; these results cast serious doubts on the assumption that morpheme and whole-word frequency effects are necessarily additive, and were in fact interpreted as evidence for two interactive systems, one involved in morpheme processing and the other involved in whole-word processing. This proposal might also be applicable to the word production system in Chinese, which would in fact nicely reconcile our results with those found by Chen and Chen [20] and by Janssen et al. [31].

Our results also demonstrate that morphology interacts with grammatical class: the difference between simple and complex words is in fact much more pronounced among nouns than among verbs. Therefore, it appears that nouns and verbs have different morphological representations and/or undergo different types of morphological processing. This result – and its theoretical interpretation – is in line with evidence obtained from studies on aphasic speakers of Indo-European languages. Shapiro, Shelton, and Caramazza [58], for example, described the case of a fluent aphasic patient who was better at producing the third-person singular form of verbs (or of nonwords inflected as verbs) than at producing the plural form of nouns (or of nonwords inflected as nouns; see also [62]). The difference in morphological processing between nouns and verbs in Chinese might be related to the specific distributional properties of Chinese compounds. In fact, the constituents that appear more frequently in nominal compounds tend to be rather high in frequency also as free-standing words; this might encourage segmentation, which would explain why compound nouns are more difficult to process than monomorphemic nouns. On the contrary, the constituents that appear more frequently in compound verbs tend to be used predominantly as bound morphemes; it is often the case, then, that the frequency of a verb compound is higher than the frequency of its constituents, which should make segmentation less likely, thus reducing the gap in difficulty between compound and simple verbs.

### 4.2. The number of strokes

The number of strokes composing the characters to be read is not a predictor of the performance of Chinese dyslexic readers. This variable was far from being significant in all analyses of simple words and compound verbs; it only turned out to be significant for the first constituent in nominal compounds, but this evidence palls given the null results on simple words and compound verbs. Our data thus confirm those reported by Law et al. [34] and are in contrast to the findings of Liu et al. [43]. The present results seem to imply that the visual complexity of the characters to be read does not impact substantially on reading accuracy; this might indicate that character recognition in Chinese is a holistic procedure based on the overall visual pattern of the whole character, rather than an analytic process that requires a detailed analysis of each stroke.

### 4.3. Lexical-semantic variables

It is not surprising that imageability influences the speakers' performance in picture naming, as this task clearly requires semantic processing of the depicted stimuli [10]. Moreover, imageability effects have been found in a number of picture naming experiments, particularly when they investigated the performance of brain-damaged individuals [12,46]. Similar results have also been obtained in studies on Chinese, both in healthy [43] and aphasic speakers [38]; this shows once again that in Chinese the semantic system is involved in picture naming. On the contrary, imageability is *not* a relevant predictor in word naming. This result is in strong contrast with the hypothesis that picture and word naming engage the same lexical-semantic pathway in Chinese because of its logographic writing system; since Chinese characters are not made up of phonologically interpretable subunits, one might in fact argue that just like people access the semantic (and phonological) representation of an object when they see its pictorial representation, similarly they might access the meaning and the phonological counterpart of a Chinese character. However, this hypothesis would also predict imageability effects in word naming, which was not found in the present study.

The inconsistent effect of imageability might be accounted for by assuming that different types of conceptual knowledge are activated when looking at a picture and looking at a character. A drawing usually activates visual semantic knowledge, whereas a character may activate lexical, functional and abstract semantic knowledge from the earliest processing phase; this would predict imageability effects predominantly in the former case, as observed in the present study. Alternatively, it could be suggested that our Chinese aphasic patients were reading along a non-semantic route. This would be in agreement with the results reported by Bi et al. [10], who described a patient with severe lexical-semantic impairment (as shown by his several semantic errors in word-to-picture matching), but spared word naming (where no semantic errors were observed). Quite intriguingly, this patient could easily read aloud words that he could not match to the corresponding pictures. These results – and those reported in the present study – suggest that reading in Chinese is also based on a dual-route system, where characters are read both by accessing their meaning (i.e., involving the conceptual system) and through a conceptually-blind procedure that bypasses the lexical-semantic store (see also [35,66]). Our data do not address the question of whether non-semantic reading takes the form of a direct association between written and spoken words (similar to the direct route of reading described in alphabetic languages), or rather of a sub-lexical routine whereby words are read on the basis of their phonetic component [10].

The proposal that reading aloud in Chinese is not necessarily mediated by the semantic system is also supported by psycholinguistic data. In a recent study, Verdonschot, Heij, and Schiller [63] carried out a picture-word interference task where healthy Chinese readers were equally fast in reading aloud words when these were superimposed on semantically related vs. unrelated pictures. It is difficult to explain these results without hypothesizing that the participants were reading aloud words non-semantically. Interestingly, when the same subjects were asked to name the pictures – rather than the written words – the typical picture-word interference effect emerged, thus indicating that the lack of semantic effect in the reading task was not due to some particular aspect of the items/subjects studied in this experiment, but was indeed due to the fact that participants were reading via a non-semantic route.

Age of acquisition seems to play the same role in reading as played by Imageability in picture naming; this result is consistent with findings recorded in the previous literature in English and in Chinese [27,38]. The nature of the AoA effect has been debated for years. Lewis [40], for example, suggested that both frequency and AoA effects depend on the total number of times that a word has been encountered in life; words acquired in the early childhood are likely to be processed (heard, read, written, or articulated) more often in someone's life than words acquired later, and thus their processing becomes faster and more accurate. Perhaps more relevantly for the present work, Barry and Gerhand [6] suggested that AoA effects arise when retrieving lexical phonology, because words acquired early in life have "more complete" explicit representations in the phonological output lexicon than words acquired later [14]. Our data are problematic for this interpretation of AoA effects, because the phonological lexicon is addressed in both picture and word naming, but in our study the AoA effect is only observed in this latter task. In fact, other studies have found AoA effects in picture naming [68], even if with a different dependent variable (response time rather than accuracy) and a different type of analysis (linear regression rather than mixed-effects models); this indicates that indeed AoA effects may arise at the level of lexical phonology. However, our data also suggest that this might not be the whole story and AoA effects might also emerge at some processing level involved in word naming, but not in picture naming. There are two available candidates:

(i) The direct route that connects the orthographic input lexicon to the phonological output lexicon by-passing the semantic system;
(ii) A sub-lexical routine whereby characters are converted into syllables on the basis of associations between phonetic components and their dominant pronunciation.

The first option appears to be more straightforward. There is no doubt about the existence of a lexical, non-semantic route for reading in Chinese [66]; moreover, Liu et al. [43] suggest that AoA reflects the mapping between orthography and phonology along this route, which of course supports oral reading only. Also considering the frequency-based interpretation of AoA described above (but see [64] for evidence against this account of AoA), it seems plausible to suggest that associations between orthographic and phonological lexical representations are stronger when words were acquired earlier in life. This proposal would also be compatible with some data obtained in English; Zevin and Seidenberg [70,71] reported that the AoA effect in reading aloud is larger for irregular words, which lead them to suggest that this effect emerges as a consequence of arbitrary mapping between orthography and phonology in the lexical network (the Arbitrary Mapping Hypothesis).

However, the second alternative cannot be discarded. Although the existence of a sub-lexical routine in Chinese has been questioned [22] on the basis of the fact that only 25% of the Chinese written words can be read correctly on the basis of their phonetic component, there is evidence that something similar to the GPC route in alphabetic languages may emerge occasionally in Chinese dyslexic patients [10]. Weekes and Chen [65], for example, have described patients with surface dyslexia who read aloud regular words better than irregular words and, perhaps more surprisingly, made errors on irregular words by producing the syllable corresponding to the dominant pronunciation of their phonetic component (LARC errors) [56]. These results could be explained in terms of a lexical (non-semantic) route for the phonetic components that are free-standing words in themselves. In this case, the phonetic component might activate its corresponding entry in the orthographic input lexicon and, subsequently, in the phonological output lexicon [74]; when the contribution of the semantic reading route is severely reduced and/or the frequency of the target is quite low, the pronunciation of the phonetic component might predominate over the correct pronunciation of the whole character, thus giving rise to a LARC error. This account, however, is clearly not applicable for the phonetic components that are not free-standing words [36, 37]; in these cases, there is no entry for the phonetic component in the orthographic lexicon and, thus, the syllable corresponding to the dominant reading of the phonetic component can only be activated from a sub-lexical reading route.

## 5. Conclusions

The results of the present study suggest the existence of a morphological level of representation in the Chinese word production system. Although our data do not support strong conjectures on where this level of representation should be placed (e.g., within the lexicon vs. post-lexically), they suggest that the process of morpheme selection and the process of word selection overlap in time and influence each other, explaining why previous studies failed to report morphological effects in word production experiments. Grammatical class is also shown to be a relevant factor in morphological processing, so that this latter may impinge differently on nouns and verbs. Finally, it has been shown that imageability does not influence the performance of brain-damaged individuals in word naming, thus suggesting that reading in Chinese aphasic patients may also occur via a non-semantic route; however, our data do not provide direct evidence as to whether this non-semantic route is lexical (i.e., comparable to the direct route of reading in Indo-European languages) or non-lexical (i.e., based on associations between non-freestanding phonetic components/characters and syllables).

## Acknowledgments

We thank Brendan Weekes, Na'ama Friedmann and an anonymous reviewer for their helpful suggestions on a previous draft of this manuscript. This work has been conducted by the second author in partial fulfillment of the requirements for the European Master in Clinical Linguistics (EMCL), an EU-supported Erasmus Mundus Excellence Program. This work has also benefited from a grant (PRIN 20074EKLSX_002) awarded to Claudio Luzzatti by the Italian Ministry of Education, University and Research (MIUR).
